# PERK Regulates Working Memory and Protein Synthesis-Dependent Memory Flexibility

**DOI:** 10.1371/journal.pone.0162766

**Published:** 2016-09-14

**Authors:** Siying Zhu, Keely Henninger, Barbara C. McGrath, Douglas R. Cavener

**Affiliations:** Department of Biology, Center of Cellular Dynamics, the Pennsylvania State University, University Park, Pennsylvania, United States of America; Technion Israel Institute of Technology, ISRAEL

## Abstract

PERK (EIF2AK3) is an ER-resident eIF2α kinase required for memory flexibility and metabotropic glutamate receptor-dependent long-term depression, processes known to be dependent on new protein synthesis. Here we investigated PERK’s role in working memory, a cognitive ability that is independent of new protein synthesis, but instead is dependent on cellular Ca^2+^ dynamics. We found that working memory is impaired in forebrain-specific *Perk* knockout and pharmacologically PERK-inhibited mice. Moreover, inhibition of PERK in wild-type mice mimics the fear extinction impairment observed in forebrain-specific *Perk* knockout mice. Our findings reveal a novel role of PERK in cognitive functions and suggest that PERK regulates both Ca^2+^ -dependent working memory and protein synthesis-dependent memory flexibility.

## Introduction

Working memory is the cognitive capacity to actively and temporarily maintain information for the purpose of task execution [1]. The dorsolateral prefrontal cortex in primates, which is homologous to the medium prefrontal cortex in rodents [2, 3], is essential for working memory as evidenced by lesion studies [4], electrophysiological recordings [5] and brain imaging [6, 7]. At the cellular level, sustained neuronal firing was observed during the delay period of working memory, which is now considered an important neuronal correlate of working memory [8]. The molecular mechanisms underlying working memory have been studied extensively in recent years, and it has been shown that intracellular Ca^2+^ signaling as stimulated by muscarinic acetylcholine or metabotropic glutamate receptor (mGluR) is critical for working memory [9–14].

PERK, an eIF2α kinase, is well known for its role in eIF2α-dependent protein synthesis and translational control. Upon activation PERK phosphorylates the α subunit of the translation initiation factor eIF2, which can subsequently modulate protein translation in two opposite ways: repression of global protein synthesis [15] and induction of translation of specific genes including CREB2/ATF4 [16]. Since both de novo protein synthesis and CREB2 are key regulators of long-term memory storage [17, 18], PERK’s role in protein synthesis-dependent cognition has been comprehensively studied, where it has been shown that PERK is required for normal flexibility in learning and memory [19] and mGluR-dependent long-term depression [20].

Besides its role in cognition, the function of PERK has been most extensively studied in the pancreatic insulin-secreting β-cells where it regulates cell proliferation, proinsulin trafficking through the secretory pathway, and insulin secretion [21, 22]. Unexpectedly the mechanism of PERK-dependent regulation of insulin secretion was found to be independent of eIF2α phosphorylation and protein synthesis. By acutely inhibiting PERK kinase activity using a newly available pharmacological inhibitor, it was discovered that PERK regulates Ca^2+^ dynamics in β-cells [23], which underlies glucose-stimulated insulin secretion. Considering the critical role of Ca^2+^ signaling in learning and memory, we hypothesized that PERK might regulate working memory, which is independent of new protein synthesis, but is largely driven by Ca^2+^ dynamics. We show herein that PERK regulates working memory. Moreover, pharmacological PERK inhibition in wild-type mice mimics the memory flexibility impairment observed in *Perk* knockout mice. These findings illustrate a novel role of PERK in cognitive function, and suggest that PERK regulates both Ca^2+^ dynamics-dependent working memory and protein synthesis-dependent memory flexibility.

## Materials and Methods

### Mouse strains

*BrPerk* KO mice were generated by crossing *Perk-*floxed mice[24] with *αCaMKII-Cre* mice T29-1 strain [25] in C57BL/6J background. Adult *BrPerk* KO mice and their wild-type littermates aged 3–7 months old were used to ensure the maximum knockdown of PERK in the forebrain [26]. Wild-type mice used in the pharmacological PERK inhibition experiments were 3 month old, and in C57BL/6J background (purchased from the Jackson laboratory). All the animal procedures were reviewed and approved by the Institutional Animal Care and Use Committee at Penn State University (IACUC# 43379).

### Brain tissue collection

To compare PERK knockdown efficiency in different brain regions, *BrPerk* KO mice and their wild-type littermates were euthanized by CO_2_ inhalation in accordance with the IACUC protocol approved by Penn State University. Different brain regions including prefrontal cortex, hippocampus and cerebellum were isolated for western blot analysis.

### Western blot analysis

Protein lysates from different brain regions and whole cells were prepared using RIPA buffer with 1X protease inhibitor and 1X phosphatase inhibitor cocktails from Sigma. Denatured protein samples were generated by boiling in 2X Laemmli buffer for 5 min. NuPAGE 4–12% Bis-Tris Midi Gel (Thermo Fisher Scientific) was used for electrophoresis. To enable the comparison of PERK knockdown efficiency in different brain regions, protein quantification was performed on protein lysates from brain tissue using Peirce BCA protein assay kit (Thermo scientific, # 23227), and 50μg protein per sample was loaded for western blot. The following primary antibodies were used in western blot analysis: monoclonal anti-PERK produced in rabbit (1:500, cell signaling, #3192), monoclonal anti-β-actin produced in mouse (1:1000, GenScript, A00702), monoclonal anti-p-PERK produced in rabbit (1:500, cell signaling, #3179), polyclonal anti-eIF2α [pS^52^] produced in rabbit (1:1000, Invitrogen, 44728G), monoclonal anti-α-tubulin produced in mouse (1:1000, Sigma, T5168).

### Gavage administration of PERK inhibitor GSK2606414

GSK2606414 was suspended in vehicle (0.5% HPMC+0.01% tween-80 in water with pH 4.0) at the concentration of 15mg/ml. Mice were gavaged with GSK2606414 at the dosage of 150mg/kg body weight or equivalent amount of vehicle during the experiment.

### Spontaneous alternation Y-maze task

Spontaneous alternation Y-maze task was performed as described [27]. Each mouse was put in the middle of a symmetrical Y maze with three identical arms (arms 35 cm long X 16 cm high) and given free access to the arms during an 8 min period. The test was video-recorded and the results including percentage alternation and number of arms entered were blindly analyzed later. The first 2 min were for habituation and the subsequent 6 min were analyzed for the sequence of entrance. An entry was recorded only when all of the animal’s limbs were within the arm. Percentage alternation was calculated as follows: the number of triads containing entries into three arms/maximum possible alternations (total number of arms entered-2) X 100. The chance level for the alternation rate is 22.2% [28].

### Reference memory test using radial-arm water maze

Reference memory was measured in radial-arm water maze, which was prepared similar to what has been previously described [29]. A circular pool with an interior diameter of 92 cm and a depth of 63 cm was used. Six identical, black, plastic 60° bended, V-shaped inserts with 45 cm in height were put into the pool to form six arms (30 cm long X 10 cm wide). The pool was filled with water made opaque by mixing with non-toxic white paint so that the platform was invisible. Visual cues were put on the conjunction of every other V-shaped insert to help orient the mouse.

Each mouse was given 8 trials per day to locate a hidden escape platform placed at the end of a fixed arm. In each trial, the animal was released from a starting arm, and given 60 s to locate the hidden platform. Once the animal got onto the platform, it was immediately taken out as a positive reward. The platform stayed in the same arm during the experiment, but the starting arm was changed every trial to avoid forming the swimming pattern. In the first 12 trials of training, the platform was altered between visible and invisible to accelerate the initial training process. The number of errors (number of wrong arms entered before getting onto the platform) was manually recorded throughout the experiment. Wild-type animals typically take 4 to 5 days to meet the criterion that they make less than 1 error on average in a trial block (4 trials per block) before locating the platform.

### The delay match-to-place task using radial-arm water maze

We performed the delay match-to-place task in the same radial-arm water maze described above. The test was consisted of two days, day 1 for training and day 2 for testing. On each day, there were five sessions for each mouse. A session consisted of a location trial and a match trial, which both lasted 120 s. In the location trial, a hidden platform was placed at the end of one arm and the animal was released from the starting arm. If the mouse successfully got onto the platform, it would be quickly taken out as a positive reward. If the mouse failed to locate the platform within the time, it would be guided onto the platform and placed on it for 10 s before being taken out. 5 s after the location trial, the mouse was released again from the starting arm to start the match trial, with the platform staying in the same location. The escape latency (time taken to get onto the platform) and the number of errors (number of wrong arms entered before getting onto the platform) were manually recorded throughout the experiment. The position of the platform was changed between sessions, while the starting arm remained the same (If the six arms were labeled as A-F clockwise, the platform was placed in different arms with the order of B-D-F-C-E, with A being the starting arm).

### Fear extinction test

The fear extinction test was carried out in the pre-pulse inhibition box from Panlab. The mice were fear conditioned on day 0 by 5 pairs of auditory conditional stimulus (85dB white noise lasting for 30 s) and unconditional stimulus (0.65mA foot-shock for 2 s) in context A (the chamber was cleaned with 1% acetic acid solution and the light was turned on in both chamber and test room). There was a 1 min interval between each pair of stimuli. Fear extinction was performed 24 hrs after fear conditioning in an altered environment context B (the chamber was cleaned with 70% ethanol, both chamber light and test room light were turned off with red fluorescent lights on as the replacement). During the extinction session, animals received 15 consecutive presentations of auditory conditional stimulus (85dB white noise lasting for 30 s) without foot-shock everyday for 2–3 days. There was a 1 min-interval between each auditory stimulus.

### Mice used in behavior experiments

In the behavior experiments performed on *BrPerk* KO mice, different groups of mice were used for each experiment. In the behavior experiments performed on pharmacologically PERK-inhibited mice, the animals used in the spontaneous alternation Y-maze task were further subjected to either short-term PERK inhibition or long-term PERK inhibition fear extinction test. Animals used in one fear extinction test were not used in the other to avoid residual effect.

### Primary neuronal culture

Mouse primary cortical neurons were prepared as previously described [30]. Briefly, cerebral cortex from day 0 wild-type pups was isolated and the cortex from each pup was dissociated in 5ml 0.05% trypsin-EDTA with DNase1 for 30 min at 37 C°(5% CO^2^). The tissue was then washed twice with HBSS containing 10% FBS, and mechanically triturated in 2ml neuronal medium with DNase1. 8ml fresh neuronal medium was added after trituration and the cells were collected by centrifugation at 120Xg for 5 min. The cells were re-suspended in 2ml fresh neuronal medium and plated on glial-coated 12mm glass coverslips in a 24-well plate at the density around 150,000 cells per well. The neuronal medium used was the MEM based medium with 5% FBS (GEMINI bio-products), 2% B27 (Invitrogen), 1mM L-Glutamine (Gibco), 20mM D-Glucose, 2μM Ara–C, 40 units/ml penicillin, 40μg/ml streptomycin and 100ng/ml Amphotericin B. Final pH was adjusted to 7.4 with NaHCO3 (100mg/500ml). Total medium was changed on DIV 1 and 50% of the medium was changed on DIV 3 and DIV 5. DIV 14–21 neurons were used for western blot analysis.

## Results

### Forebrain-specific *Perk* knockout mice are impaired in spontaneous alternation Y-maze task

**Fig 1:**
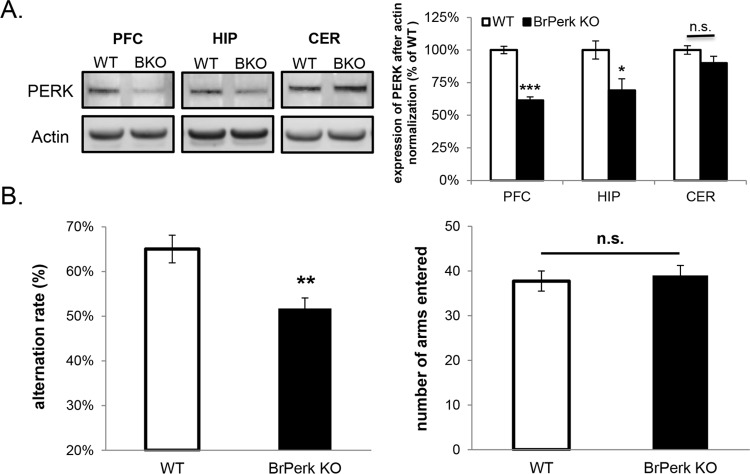
*BrPerk* KO mice are impaired in spontaneous alternation Y-maze task. (A) Western blot analysis showing substantial knockdown of PERK in the prefrontal cortex (PFC) of *BrPerk* KO mice. PERK was also significantly knocked down in the hippocampus (HIP) but not in the cerebellum (CER). (BKO: *BrPerk* KO; *** p<0.001, * p<0.05, n.s. not significant, two-tailed student’s t-Test; n = 5 for each genotype). (B) Spontaneous alternation Y-maze task. *BrPerk* KO mice exhibited a lower alternation rate compared to the wild-type littermates, indicating their poor spatial working memory (WT n = 15, *BrPerk* KO n = 22; ** p<0.01, two-tailed student’s t-Test). No difference was observed in total number of arms entered (n.s. not significant, two-tailed student’s t-Test), indicating similar motor ability and curiosity for the environment between two genotypes. Impaired spontaneous alternation was observed in *BrPerk* KO group in both genders. The figures represent pooled results of both genders.

To study the function of PERK in learning and memory, we generated forebrain-specific *Perk* knockout (*BrPerk* KO) mice by crossing *αCamKII-Cre* with *Perk-floxed* mice previously generated in our lab [24]. The expression of Cre recombinase in the *αCaMKII-Cre* strain occurs primarily in postnatal forebrain peaking at 3 months old [26]. We therefore restricted our studies to adult *BrPerk* KO mice at least 3 months old. Previously it has been reported that Cre recombinase is expressed in both cortex and hippocampus in the *αCaMKII-Cre* strain [31, 32]. Western blot analysis of homogenates of different brain regions confirmed that the expression of PERK was substantially knocked down in the prefrontal cortex (Fig 1A), which is the major brain region involved in working memory and memory flexibility in rodents [1, 33]. Significant knockdown of PERK was also observed in the hippocampus but not in the cerebellum (Fig 1A). The small amount of residual PERK seen in *BrPerk* KO mice forebrain was likely due to PERK’s expression in glia and in non-pyramidal neurons, and to incomplete deletion of PERK in pyramidal neurons. The generation of *BrPerk* KO mice enabled us to specifically look at the function of PERK in the forebrain at adult stage without disturbing PERK’s function in other tissues or in earlier neurodevelopment.

As a specific form of memory, working memory lasts for only several seconds and does not require new protein synthesis. To determine if PERK is required for working memory, *BrPerk* KO mice were subjected to the spontaneous alternation Y-maze task, which is a spatial working memory test that is based on the willingness of rodents to explore a new environment [34]. In this task, an animal must remember the arm it had visited previously in order to alternate the arm entrance in the next trial. Thus the alternation rate is an indicator of the animal’s spatial working memory capacity. *BrPerk* KO mice exhibited significantly less alternation rate when compared to their wild-type littermates, but did not differ in the total number of arms entered (Fig 1B). These results show that *BrPerk* KO mice are impaired in spatial working memory, but exhibit similar levels of locomotion and exploratory behavior.

### *BrPerk* KO mice are impaired in the delay match-to-place task

**Fig 2:**
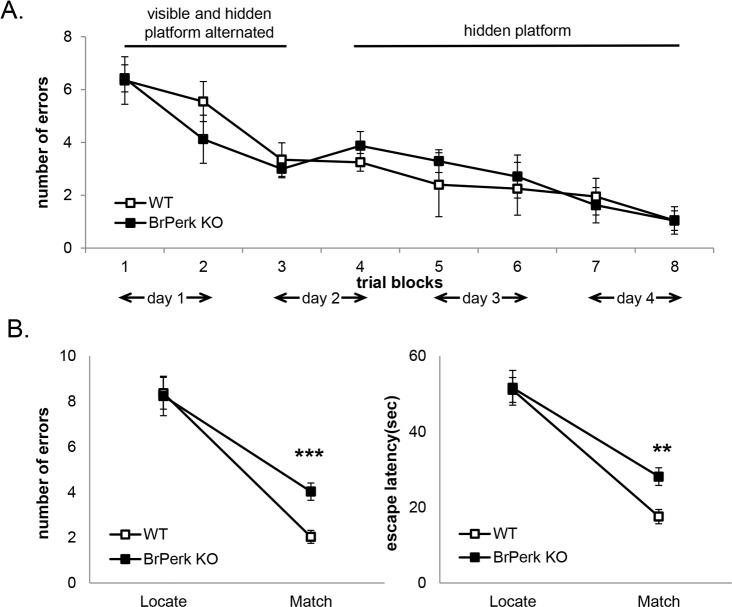
*BrPerk* KO mice are impaired in the delay match-to place working memory task. (A) Spatial reference memory test in radial-arm water maze. *BrPerk* KO mice exhibited normal reference memory in a 4 day reference platform task in radial-arm water maze (WT n = 5, *BrPerk* KO n = 6). Each mouse was given 8 trials per day during the 4 day task. A trial block represents the average number of errors of 4 trials. Only male mice were used in the experiment. (B) The delay match-to-place task in radial-arm water maze. *BrPerk* KO mice made more errors and required longer escape latency than their wild-type littermates to locate the hidden platform in the match trial (WT n = 16, *BrPerk* KO n = 20; *** p<0.001, ** p<0.01, two-tailed student’s t-Test). Impaired spatial working memory was observed in *BrPerk* KO group in both genders. The figures represent pooled results of both genders.

To further examine the working memory capacity in *BrPerk* KO mice, we challenged the *BrPerk* KO mice with a more complex working memory test: the delay match-to-place task using radial-arm water maze. Successful accomplishment of this task requires good spatial reference memory and working memory. Previously it has been shown that *BrPerk* KO mice possess normal hippocampus-dependent spatial reference memory when tested in Morris water maze [19]. We repeated the spatial reference memory test in radial-arm water maze, and confirmed that there is no difference between *BrPerk* KO mice and their wild-type littermates (Fig 2A). After the reference memory test, *BrPerk* KO mice were subjected to a delay match-to-place test in radial-arm water maze. In this test, the mouse was given five sessions each day, with the location of the hidden platform changed between successive sessions. Each session consisted of two trials, a location trial followed by a match trial. A mouse with intact working memory would remember the location of the hidden platform in the location trial, and use visual cues to orient itself, thus displaying substantial savings of escape latency in the match trial [35]. The *BrPerk* KO mice made more errors and required longer escape latency in locating the platform in match trials compared to their wild-type littermates (Fig 2B), suggesting an impairment in working memory.

### Pharmacologically PERK-inhibited mice are impaired in spontaneous alternation Y-maze task

**Fig 3:**
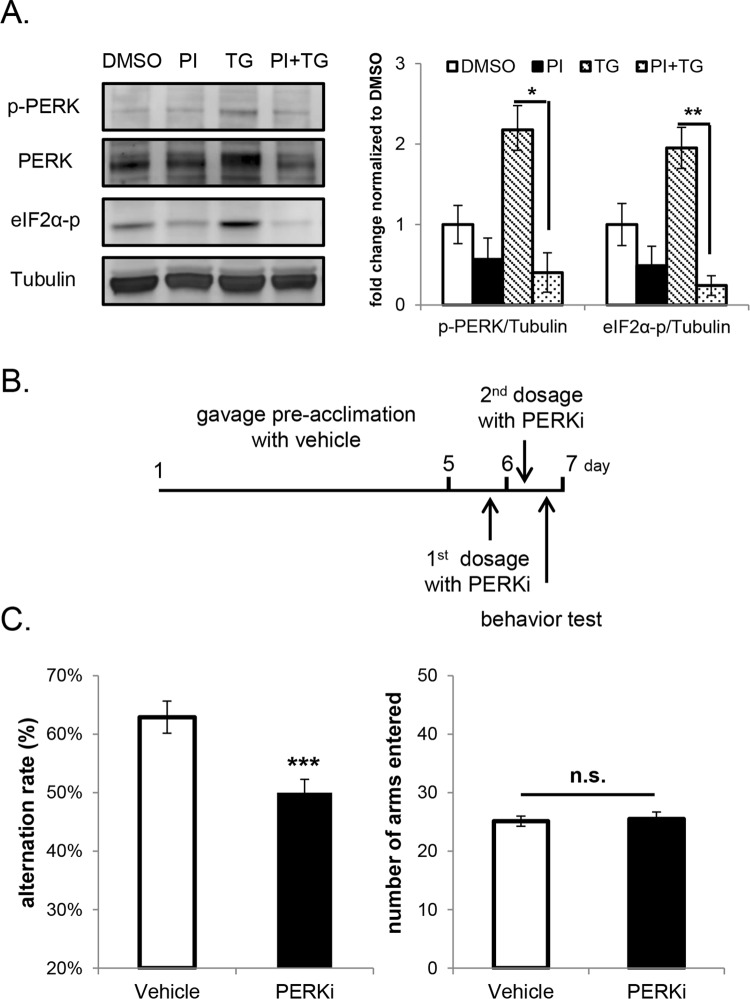
WT mice with short-term PERK inhibition are impaired in Y-maze spontaneous alternation. (A) Western blot analysis showing PERK inhibitor (PI) pretreatment prevented thapsigargin (TG)-induced phosphorylation of PERK and its substrate eIF2α in primary cortical neurons (one-way ANOVA followed by Bonferroni’s post-hoc test, * p<0.05, ** p<0.01). Cells were pretreated with 500 nM PI or DMSO for 15 min followed by co-treatment with or without 100 nM TG for 30 min. The phosphorylation level of PERK is shown by p-PERK blot, and the shifted band in PERK blot due to the dimerization and auto-phosphorylation of PERK after its activation. 3 replicates were included in the experiment and the quantification was performed on pooled results. (B) Schematic of PERK inhibitor treatment in spontaneous alternation Y-maze task. (C) Spontaneous alternation Y-maze task. Short-term PERK-inhibited mice showed a lower alteration rate compared to vehicle group (Vehicle n = 17, PERKi n = 18; ***p<0.001, two-tailed student’s t-Test). No difference was observed in the total number of arms entered (n.s. not significant, two-tailed student’s t-Test). Only male mice were used in the experiment.

To investigate if a similar behavioral impairment would occur following pharmacological inhibition of PERK activity, we systematically treated the wild-type mice with a newly developed PERK inhibitor (PERKi), GSK2606414. GSK2606414 is a highly specific inhibitor of PERK, which acts by competing for the ATP binding domain in the catalytic site [36], and has been proven to be effective in acutely inhibiting PERK’s activity in cultured cells [36]. To verify the effectiveness of PERKi in vitro, primary cortical neurons were pretreated with 500nM PERKi for 15 min followed by activation of PERK with 100nM thapsigargin for 30 min. Thapsigargin is a non-reversible SERCA pump inhibitor [37], that activates the PERK kinase activity by causing ER calcium depletion [38]. 15 min-pretreatment of 500nM PERKi inhibited thapsigargin-induced PERK activation and eIF2α phosphorylation, demonstrating that the PERKi effectively inhibits PERK’s activity in primary neurons (Fig 3A). Due to the low level of PERK activation in mice brain at steady state (preliminary data), we were unable to validate the efficacy of PERKi in vivo by western blot analysis. On the other hand, the efficacy of PERKi in vivo has been validated by others using liquid chromatography-tandem mass spectrometry measurements, and it was demonstrated that orally administered GSK2606414 penetrates the blood-brain barrier in mice [39].

Oral gavage is the only effective means of administering the PERKi, however oral gavage results in acute stress [40] and short-term elevation of corticosterone levels [41]. To relieve gavage-induced stress, mice were acclimated to gavage administration by dosing with vehicle daily for 5 days prior to the administration of the inhibitor. After gavage acclimation, the animals were administered with 2 doses of PERKi or vehicle, and their working memory was assessed in spontaneous alternation Y-maze task 8 hours after the 2^nd^ dose of PERKi (Fig 3B). The selection of 8 hours allowed dissipation of gavage-induced stress while retaining sufficient inhibitor levels in circulation (preliminary data). Since saturating concentrations of GSK2606414 in mice brains have been reported 14 hours after 2 doses of compound administration at the concentration of 50mg/kg [39], a sufficient amount of the PERKi should be present in the brain 8 hours after 2 doses of GSK2606414 administration at the concentration of 150mg/kg. The PERK-inhibited mice exhibited a lower alternation rate compared to the vehicle controls (Fig 3C), suggesting the impairment of spatial working memory. Similar to genetic *Perk* ablation, no difference was seen in the total number of arms entered between the PERKi and vehicle groups (Fig 3C). However mice received gavage treatment showed significantly less total arm entrance when compared to the mice with no gavage treatment (number of arms entered in Fig 1B and Fig 3C were compared; p<0.001, one-way ANOVA followed by Bonferroni’s post-hoc test), which suggests the possible impairment in animal performance due to the residual gavage-induced stress.

### Pharmacological PERK inhibition mimics fear extinction impairment in *BrPerk* KO mice

Previous studies reported that *BrPerk* KO mice are impaired in protein synthesis-dependent memory flexibility, as shown by Y-water maze reversal task and fear extinction task [19]. We tested the memory flexibility of the *BrPerk* KO using the Y-water maze reversal task and confirmed this result (S1 Fig). To examine whether pharmacological PERK inhibition could mimic the memory flexibility impairments observed in genetic *Perk* knockout mice, we measured fear extinction memory in wild-type mice orally administered with PERKi. We chose to test fear extinction memory because this task requires less precise cognition compared to Y-water maze reversal task, and thus is less likely to be confounded by gavage-induced stress.

**Fig 4:**
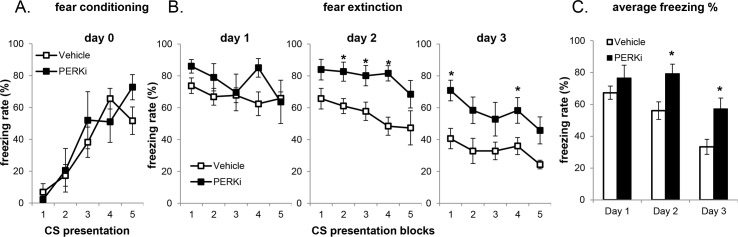
WT mice with short-term PERK inhibitor gavage administration are impaired in fear extinction. (A) Fear conditioning on day 0. No difference was observed between treatments. (B) Fear extinction over 3 days (3 CS presentations/block; Vehicle, n = 6; PERKi, n = 5; * p<0.05, two-tailed student’s t-Test). PERK-inhibited mice exhibited higher freezing rate over 3 days’ extinction session (p<0.001, non-parametric paired sign test). Only male mice were used in the experiment. (C) Average freezing rate over 3 days’ extinction session. Short-term PERK-inhibited mice exhibited higher average freezing rate on the 2^nd^ and 3^rd^ day of extinction session, indicating their impairment in fear extinction (* p<0.05, two-tailed student’s t-Test).

Fear extinction memory was measured in wild-type mice which received short-term or long-term PERKi treatment. In the short-term PERKi treatment group, the mice were first acclimated to gavage for 5 days by daily vehicle administration, followed by 2 doses of PERKi or vehicle before the behavioral test (Fig 3B), and 1 dose daily during the course of the experiment. Short-term PERK inhibition did not affect fear conditioning (Fig 4A). However, whereas the vehicle group started to exhibit a progressive decline of freezing rate with repeated exposure to CS on the 2^nd^ and 3^rd^ day of extinction session, the freezing rate of PERKi group remained elevated (Fig 4B and 4C). This result mimics the extinction impairment observed previously in *BrPerk* KO mice. It is noteworthy that neither group exhibited significant fear extinction on day 1. This was likely the result of gavage-induced stress, because in our initial trials when mice were not first acclimated to gavage neither vehicle nor PERKi group showed significant decline in the freezing rate over 2 days’ extinction training (S2 Fig).

**Fig 5:**
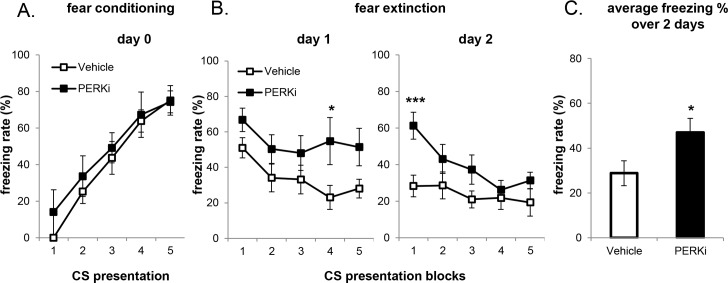
WT mice with long-term PERK inhibitor gavage administration are impaired in fear extinction. (A) Fear conditioning on day 0. No difference was observed between treatments. (B) Fear extinction over 2 days (3 CS presentations/block; Vehicle n = 10, PERKi n = 8, * p<0.05, *** p<0.001, two-tailed student’s t-Test). Long-term PERK-inhibited mice exhibited delayed fear extinction over the 2 days (p<0.01, non-parametric paired sign test). Only male mice were used in the experiment. (C) Average freezing rate over 2 days’ extinction session. PERK-inhibited mice exhibited higher average freezing rate over 2 days’ extinction session, indicating their impairment in fear extinction (* p<0.05, two-tailed student’s t-Test).

In the long-term PERKi treatment group, mice were orally administered with inhibitor or vehicle for 2 weeks prior to the behavioral test. The mice in this experiment were not acclimated to gavage because of long-term gavage administration. As with the short-term PERK inhibition, no difference in fear conditioning was observed between treatments (Fig 5A). With longer inhibitor treatment, while both groups exhibited significant fear extinction on day 1, PERK-inhibited mice exhibited delayed extinction over the 2 days (Fig 5B and 5C).

## Discussion

Previous study of PERK’s function in the brain has focused on protein synthesis-dependent cognitive abilities where it has been demonstrated that PERK is required for memory flexibility and normal expression of mGluR-dependent long-term depression via eIF2α-dependent protein translational control. Two other eIF2α kinases, GCN2 and PKR, have also been shown to be involved in protein synthesis-dependent memory regulation [42, 43]. Motivated by the recent discovery that PERK acutely regulates Ca^2+^ dynamics in β-cells [23] and primary neurons (preliminary data), which is independent of phosphorylation of eIF2α and protein translational control, we explored the possibility that PERK may regulate Ca^2+^ dynamics-dependent working memory. We found that *BrPerk* KO mice are impaired in working memory, as shown by spontaneous alternation Y-maze task and the delay match-to-place task. Since over the course of development the genetic *Perk* knockout mice may have acquired a compensatory mechanism that could potentially confound the interpretation of underlying mechanisms, we examined the effects of pharmacologically ablating PERK activity in wild-type mice by oral administration of a specific and potent PERK inhibitor. PERK-inhibited mice exhibited significant impairment in working memory in the spontaneous alternation Y-maze task. Moreover, oral administration of PERKi mimics the fear extinction deficit observed in forebrain-specific *Perk* knockout mice. Together, these results suggest that the behavior phenotypes observed in *BrPerk* KO mice are attributable to the absence of PERK rather than to a developmental compensatory response.

Spontaneous alternation Y-maze task and the delay match-to-place task in radial-arm water maze were used to test spatial working memory in this study. Y-maze spontaneous alternation occurs because of rodents’ natural exploratory behavior, which causes them to alternate spontaneously in the Y-maze, at the rate significantly higher than that could be due to chance [34]. Although the neuronal mechanisms underlying spontaneous alternation are still unclear, this test has been widely used to measure spatial working memory, because it does not require excessive animal handling and measures subject’s locomotion in the meantime [44]. In addition to spontaneous alternation Y-maze task, we also subjected *BrPerk* KO mice to the delay match-to-place task. The delay match-to-place task is a robust behavioral test, but it is also a more complex test for measuring spatial working memory. Since the initial introduction by Morris and coworkers [45], modifications have been made to simplify the procedure without reducing the maze’s complexity [35, 46]. We performed the delay match-to-place task in radial-arm water maze, which preserves the spatial complexity of the Morris water tank, but simplifies scoring procedure with the benefit of a defined swim path.

The neuronal mechanisms underlying PERK’s regulation of working memory is unknown. We speculate that PERK regulates working memory via changes in Ca^2+^ dynamics based upon PERK’s demonstrated function in regulating Ca^2+^ dynamics in β-cells. Our lab has shown that PERK regulates ER Ca^2+^ re-uptake and cytosol Ca^2+^ influx from extracellular space in β-cells, which underlies normal glucose-stimulated insulin secretion [23]. Similarly, in neurons, it has been shown that metabotropic receptor-coupled intracellular Ca^2+^ ([Ca^2+^]_i_) rise is important for working memory in several ways. Firstly, the induced [Ca^2+^]_i_ rise is known to activate several Ca^2+^-dependent proteins including phosphatase calcineurin, kinase CaMKII and PKC, all of which have been shown to regulate working memory capacity [47]. Moreover, metabotropic receptor-coupled [Ca^2+^]_i_ can directly affect intrinsic neuronal properties. For example, it has been shown that mGluR mediated [Ca^2+^]_i_ regulates SK channel-dependent neuronal hyperpolarization and TRPC-dependent neuronal depolarization, indicating the amplitude of [Ca^2+^]_i_ can influence neuronal excitability [14, 48]. In our preliminary studies (unpublished), we have observed that acute PERK inhibition impairs metabotropic receptor-coupled [Ca^2+^]_i_ due to impaired receptor-operated Ca^2+^ influx in primary cortical neurons, which suggests that these pathways may also underlie the neuronal mechanism of PERK’s regulation of working memory in vivo.

Fear extinction is a form of inhibitory learning, a process by which a pre-conditioned fear response is extinguished after repeated exposure to neutral conditioned stimulus without the aversive stimulus. Previously it has been shown that *BrPerk* KO mice are impaired in fear extinction, which is associated with reduced eIF2α-dependent ATF4 expression [19]. Here we showed that pharmacological PERK inhibition mimics the fear extinction impairment observed in *BrPerk* KO mice, which suggests that the behavior phenotype observed in *BrPerk* KO mice is attributable to the absence of PERK, and confirms PERK’s role in regulating memory flexibility in addition to working memory.

Although different molecular mechanisms have been suggested underlying working memory and memory flexibility, both cognitive functions are considered as core components of executive function, regulated in different sub-regions in prefrontal cortex, but modulated by similar neurotransmitters [1, 33]. Thus, the possibility remains that the working memory and memory flexibility deficits observed in *BrPerk* mice may be due to the impairment in a common pathway that is normally regulated by PERK.

Based on cell culture experiments, PERK was initially identified as an effector of unfolded protein and ER stress response [15, 38, 49] and from studies it was speculated that the function of PERK in whole organisms was to temporarily repress protein synthesis in order to relieve the client protein load in the ER. However subsequent studies in *Perk* KO mice, which display an array of dysfunctions, failed to find evidence in support of this hypothesis [21, 50]. Rather we found that PERK plays important developmental and physiological regulatory functions that are unrelated to stress throughout the body [21, 22, 50, 51]. In this study, we investigated PERK’s role in cognition, and showed for the first time that PERK regulates new protein synthesis-independent working memory. This discovery reveals a novel physiological role of PERK in cognitive function, which is unrelated to stress responses. Moreover, eIF2α dephosphorylation has been shown to be critical for late phase long-term potentiation (L-LTP) elicitation and long-term memory (LTM) storage, as genetic reduction of eIF2α phosphorylation by knockdown of eIF2α kinases GCN2 [42] or PKR [43], or single allele phosphorylation site mutation of eIF2α (Serine to Alanine at phosphorylation site Ser51) [52] lowers the threshold for L-LTP and facilitates LTM, while pharmacological promotion of eIF2α phosphorylation impairs L-LTP and LTM [52]. On the other hand, forebrain-specific *Perk* knockout mice exhibit normal LTP and LTM [19, 20], but impaired memory flexibility [19]. The significant phenotype difference between *Perk* knockout mice and other mice models with reduced eIF2α phosphorylation suggest that PERK may possess additional regulation of cognition that is eIF2α-independent. The discovery of PERK’s regulation over Ca^2+^ dynamics-dependent working memory supports the above hypothesis. Further study is needed to elucidate the specific pathways that participate in PERK’s regulation of the different cognitive functions.

## Supporting Information

S1 Fig *BrPerK* KO mice exhibit impaired memory flexibility in Y-maze reversal task. *BrPerk* KO mice exhibited normal spatial learning in the training session on day 1, and normal long-term memory in the test session on day 2 (WT: 4 out of 4 passed the test; *BrPerk* KO: 4 out of 5 passed the test). In the reversal session on day 2, when the platform was switched to the opposing arm, *BrPerk* KO mice exhibited impaired memory flexibility, as illustrated by the lower percentage of correct arm choice/block (WT n = 4; *BrPerk* KO n = 4; ** p<0.01. two-tailed student’s t-Test; 4 trials were included per block in the training and reversal session, 2 trials were performed in the test session). A retraining trial was performed between the third and fourth trial block in the reversal session, and the mice who never located the platform in its new location were guided onto it.
(TIF)

S2 Fig Acute gavage administration impairs fear extinction learning. For mice received acute gavage administration without gavage acclimation, while both vehicle and PERKi group acquired conditioned fear on day 0, neither group exhibited fear extinction over the 2 day extinction training (Vehicle n = 10; PERKi n = 9).
(TIF)

S1 File Material and Method. Material and method for Y-water maze reversal task.
(DOCX)

## References

[1] Khan ZU, Muly EC. Molecular mechanisms of working memory. Behavioural brain research. 2011;219(2):329–41. 10.1016/j.bbr.2010.12.039 21232555

[2] Kolb B, Tees RC. The Cerebral cortex of the rat. Cambridge, Mass.: MIT Press; 1990. xii, 645 p. p.

[3] Guldin WO, Pritzel M, Markowitsch HJ. Prefrontal cortex of the mouse defined as cortical projection area of the thalamic mediodorsal nucleus. Brain Behav Evol. 1981;19(3–4):93–107. 7326577 10.1159/000121636

[4] Jacobsen CF. Functions of frontal association area in primates. Archives of Neurology and Psychiatry. 1935; 33:558–569.

[5] Fuster JM, Alexander GE. Neuron activity related to short-term memory. Science. 1971;173(3997):652–4. 4998337 10.1126/science.173.3997.652

[6] D'Esposito M, Detre JA, Alsop DC, Shin RK, Atlas S, Grossman M. The neural basis of the central executive system of working memory. Nature. 1995;378(6554):279–81. 7477346 10.1038/378279a0

[7] Cohen JD, Perlstein WM, Braver TS, Nystrom LE, Noll DC, Jonides J, et al Temporal dynamics of brain activation during a working memory task. Nature. 1997;386(6625):604–8. 9121583 10.1038/386604a0

[8] Goldman-Rakic PS. Cellular basis of working memory. Neuron. 1995;14(3):477–85. 7695894 10.1016/0896-6273(95)90304-6

[9] Penetar DM, McDonough JH Jr. Effects of cholinergic drugs on delayed match-to-sample performance of rhesus monkeys. Pharmacology, biochemistry, and behavior. 1983;19(6):963–7. 6657730 10.1016/0091-3057(83)90399-4

[10] Granon S, Poucet B, Thinus-Blanc C, Changeux JP, Vidal C. Nicotinic and muscarinic receptors in the rat prefrontal cortex: differential roles in working memory, response selection and effortful processing. Psychopharmacology. 1995; 119:139–144. 7659760 10.1007/BF02246154

[11] Hayashi K, Yoshihara T, Ichitani Y. Involvement of hippocampal metabotropic glutamate receptors in radial maze performance. Neuroreport. 2007; 18:719–723. 17426606 10.1097/WNR.0b013e3280d9e880

[12] Mikami A, Masuoka T, Yasuda M, Yamamoto Y, Kamei C. Participation of cholinergic system in memory deficits induced by blockade of hippocampal mGlu(1) receptors. European journal of pharmacology. 2007;575(1–3):82–6. 17678890 10.1016/j.ejphar.2007.07.010

[13] Egorov AV, Hamam BN, Fransen E, Hasselmo ME, Alonso AA. Graded persistent activity in entorhinal cortex neurons. Nature. 2002;420(6912):173–8. 12432392 10.1038/nature01171

[14] Hagenston AM, Fitzpatrick JS, Yeckel MF. MGluR-mediated calcium waves that invade the soma regulate firing in layer V medial prefrontal cortical pyramidal neurons. Cerebral cortex. 2008;18(2):407–23. 17573372 10.1093/cercor/bhm075 PMC3005283

[15] Harding HP, Zhang Y, Bertolotti A, Zeng H, Ron D. Perk is essential for translational regulation and cell survival during the unfolded protein response. Molecular cell. 2000;5(5):897–904. 10882126 10.1016/s1097-2765(00)80330-5

[16] Vattem KM, Wek RC. Reinitiation involving upstream ORFs regulates ATF4 mRNA translation in mammalian cells. Proc Natl Acad Sci U S A. 2004;101(31):11269–74. 15277680 10.1073/pnas.0400541101 PMC509193

[17] Davis HP, Squire LR. Protein synthesis and memory: a review. Psychol Bull. 1984;96(3):518–59. 6096908

[18] Chen A, Muzzio IA, Malleret G, Bartsch D, Verbitsky M, Pavlidis P, et al Inducible enhancement of memory storage and synaptic plasticity in transgenic mice expressing an inhibitor of ATF4 (CREB-2) and C/EBP proteins. Neuron. 2003;39(4):655–69. 12925279 10.1016/s0896-6273(03)00501-4

[19] Trinh MA, Kaphzan H, Wek RC, Pierre P, Cavener DR, Klann E. Brain-specific disruption of the eIF2alpha kinase PERK decreases ATF4 expression and impairs behavioral flexibility. Cell reports. 2012;1(6):676–88. 10.1016/j.celrep.2012.04.010 22813743 PMC3401382

[20] Trinh MA, Ma T, Kaphzan H, Bhattacharya A, Antion MD, Cavener DR, et al The eIF2alpha kinase PERK limits the expression of hippocampal metabotropic glutamate receptor-dependent long-term depression. Learning & memory. 2014;21(5):298–304. 24741110 10.1101/lm.032219.113 PMC3994503

[21] Zhang W, Feng D, Li Y, Iida K, McGrath B, Cavener DR. PERK EIF2AK3 control of pancreatic beta cell differentiation and proliferation is required for postnatal glucose homeostasis. Cell metabolism. 2006;4(6):491–7. 17141632 10.1016/j.cmet.2006.11.002

[22] Gupta S, McGrath B, Cavener DR. PERK (EIF2AK3) regulates proinsulin trafficking and quality control in the secretory pathway. Diabetes. 2010;59(8):1937–47. 10.2337/db09-1064 20530744 PMC2911049

[23] Wang R, McGrath BC, Kopp RF, Roe MW, Tang X, Chen G, et al Insulin secretion and Ca2+ dynamics in beta-cells are regulated by PERK (EIF2AK3) in concert with calcineurin. The Journal of biological chemistry. 2013;288(47):33824–36. 10.1074/jbc.M113.503664 24114838 PMC3837125

[24] Zhang P, McGrath B, Li S, Frank A, Zambito F, Reinert J, et al The PERK eukaryotic initiation factor 2 alpha kinase is required for the development of the skeletal system, postnatal growth, and the function and viability of the pancreas. Molecular and cellular biology. 2002;22(11):3864–74. 11997520 10.1128/MCB.22.11.3864-3874.2002 PMC133833

[25] Tsien JZ, Chen DF, Gerber D, Tom C, Mercer EH, Anderson DJ, et al Subregion- and cell type-restricted gene knockout in mouse brain. Cell. 1996;87(7):1317–26. 8980237 10.1016/s0092-8674(00)81826-7

[26] Minichiello L, Korte M, Wolfer D, Kuhn R, Unsicker K, Cestari V, et al Essential role for TrkB receptors in hippocampus-mediated learning. Neuron. 1999;24(2):401–14. 10571233 10.1016/s0896-6273(00)80853-3

[27] Oakley H, Cole SL, Logan S, Maus E, Shao P, Craft J, et al Intraneuronal beta-amyloid aggregates, neurodegeneration, and neuron loss in transgenic mice with five familial Alzheimer's disease mutations: potential factors in amyloid plaque formation. The Journal of neuroscience: the official journal of the Society for Neuroscience. 2006;26(40):10129–40. 17021169 10.1523/JNEUROSCI.1202-06.2006 PMC6674618

[28] Wolf A, Bauer B, Abner EL, Ashkenazy-Frolinger T, Hartz AM. A Comprehensive Behavioral Test Battery to Assess Learning and Memory in 129S6/Tg2576 Mice. PloS one. 2016;11(1):e0147733 10.1371/journal.pone.0147733 26808326 PMC4726499

[29] Alamed J, Wilcock DM, Diamond DM, Gordon MN, Morgan D. Two-day radial-arm water maze learning and memory task; robust resolution of amyloid-related memory deficits in transgenic mice. Nature protocols. 2006;1(4):1671–9. 17487150 10.1038/nprot.2006.275

[30] Chen G, Trombley PQ, van den Pol AN. Excitatory actions of GABA in developing rat hypothalamic neurones. The Journal of physiology. 1996;494 (Pt 2):451–64. 8842004 10.1113/jphysiol.1996.sp021505 PMC1160647

[31] Madisen L, Zwingman TA, Sunkin SM, Oh SW, Zariwala HA, Gu H, et al A robust and high-throughput Cre reporting and characterization system for the whole mouse brain. Nature neuroscience. 2010;13(1):133–40. 10.1038/nn.2467 20023653 PMC2840225

[32] Yamanaka T, Tosaki A, Kurosawa M, Akimoto K, Hirose T, Ohno S, et al Loss of aPKClambda in differentiated neurons disrupts the polarity complex but does not induce obvious neuronal loss or disorientation in mouse brains. PloS one. 2013;8(12):e84036 10.1371/journal.pone.0084036 24391875 PMC3877147

[33] Logue SF, Gould TJ. The neural and genetic basis of executive function: attention, cognitive flexibility, and response inhibition. Pharmacology, biochemistry, and behavior. 2014;123:45–54. 10.1016/j.pbb.2013.08.007 23978501 PMC3933483

[34] Lalonde R. The neurobiological basis of spontaneous alternation. Neuroscience and biobehavioral reviews. 2002;26(1):91–104. 11835987 10.1016/s0149-7634(01)00041-0

[35] Runyan JD, Moore AN, Dash PK. A role for prefrontal calcium-sensitive protein phosphatase and kinase activities in working memory. Learning & memory. 2005;12(2):103–10. 15805309 10.1101/lm.89405 PMC1074327

[36] Axten JM, Medina JR, Feng Y, Shu A, Romeril SP, Grant SW, et al Discovery of 7-methyl-5-(1-{[3-(trifluoromethyl)phenyl]acetyl}-2,3-dihydro-1H-indol-5-yl)-7H-p yrrolo[2,3-d]pyrimidin-4-amine (GSK2606414), a potent and selective first-in-class inhibitor of protein kinase R (PKR)-like endoplasmic reticulum kinase (PERK). Journal of medicinal chemistry. 2012;55(16):7193–207. 10.1021/jm300713s 22827572

[37] Lytton J, Westlin M, Hanley MR. Thapsigargin inhibits the sarcoplasmic or endoplasmic reticulum Ca-ATPase family of calcium pumps. The Journal of biological chemistry. 1991;266(26):17067–71. 1832668

[38] Harding HP, Zhang Y, Ron D. Protein translation and folding are coupled by an endoplasmic-reticulum-resident kinase. Nature. 1999;397(6716):271–4. 9930704 10.1038/16729

[39] Moreno JA, Halliday M, Molloy C, Radford H, Verity N, Axten JM, et al Oral treatment targeting the unfolded protein response prevents neurodegeneration and clinical disease in prion-infected mice. Sci Transl Med. 2013;5(206):206ra138 10.1126/scitranslmed.3006767 24107777

[40] Balcombe JP, Barnard ND, Sandusky C. Laboratory routines cause animal stress. Contemp Top Lab Anim Sci. 2004;43(6):42–51. 15669134

[41] Brown AP, Dinger N, Levine BS. Stress produced by gavage administration in the rat. Contemp Top Lab Anim Sci. 2000;39(1):17–21. 11178310

[42] Costa-Mattioli M, Gobert D, Harding H, Herdy B, Azzi M, Bruno M, et al Translational control of hippocampal synaptic plasticity and memory by the eIF2alpha kinase GCN2. Nature. 2005;436(7054):1166–73. 16121183 10.1038/nature03897 PMC1464117

[43] Zhu PJ, Huang W, Kalikulov D, Yoo JW, Placzek AN, Stoica L, et al Suppression of PKR promotes network excitability and enhanced cognition by interferon-gamma-mediated disinhibition. Cell. 2011;147(6):1384–96. 10.1016/j.cell.2011.11.029 22153080 PMC3569515

[44] Hughes RN. The value of spontaneous alternation behavior (SAB) as a test of retention in pharmacological investigations of memory. Neuroscience and biobehavioral reviews. 2004;28(5):497–505. 15465137 10.1016/j.neubiorev.2004.06.006

[45] Morris RG, Hagan JJ, Rawlins JN. Allocentric spatial learning by hippocampectomised rats: a further test of the "spatial mapping" and "working memory" theories of hippocampal function. Q J Exp Psychol B. 1986;38(4):365–95. 3809580

[46] Morgan D, Diamond DM, Gottschall PE, Ugen KE, Dickey C, Hardy J, et al A beta peptide vaccination prevents memory loss in an animal model of Alzheimer's disease. Nature. 2000;408(6815):982–5. 11140686 10.1038/35050116

[47] Dash PK, Moore AN, Kobori N, Runyan JD. Molecular activity underlying working memory. Learning & memory. 2007;14(8):554–63. 17690339 10.1101/lm.558707

[48] El-Hassar L, Hagenston AM, D'Angelo LB, Yeckel MF. Metabotropic glutamate receptors regulate hippocampal CA1 pyramidal neuron excitability via Ca(2)(+) wave-dependent activation of SK and TRPC channels. The Journal of physiology. 2011;589(Pt 13):3211–29. 10.1113/jphysiol.2011.209783 21576272 PMC3145935

[49] Shi Y, Vattem KM, Sood R, An J, Liang J, Stramm L, et al Identification and characterization of pancreatic eukaryotic initiation factor 2 alpha-subunit kinase, PEK, involved in translational control. Molecular and cellular biology. 1998;18(12):7499–509. 9819435 10.1128/mcb.18.12.7499 PMC109330

[50] Wei J, Sheng X, Feng D, McGrath B, Cavener DR. PERK is essential for neonatal skeletal development to regulate osteoblast proliferation and differentiation. J Cell Physiol. 2008;217(3):693–707. 10.1002/jcp.21543 18683826

[51] Li Y, Iida K, O'Neil J, Zhang P, Li S, Frank A, et al PERK eIF2alpha kinase regulates neonatal growth by controlling the expression of circulating insulin-like growth factor-I derived from the liver. Endocrinology. 2003;144(8):3505–13. 12865332 10.1210/en.2003-0236

[52] Costa-Mattioli M, Gobert D, Stern E, Gamache K, Colina R, Cuello C, et al eIF2alpha phosphorylation bidirectionally regulates the switch from short- to long-term synaptic plasticity and memory. Cell. 2007;129(1):195–206. 17418795 10.1016/j.cell.2007.01.050 PMC4149214

