# Supplementary figures and images for: PERK Regulates Working Memory and Protein Synthesis-Dependent Memory Flexibility
Source: PLoS One. 2016 Sep 14;11(9):e0162766. doi: 10.1371/journal.pone.0162766 (PMC5023101; doi:10.1371/journal.pone.0162766)

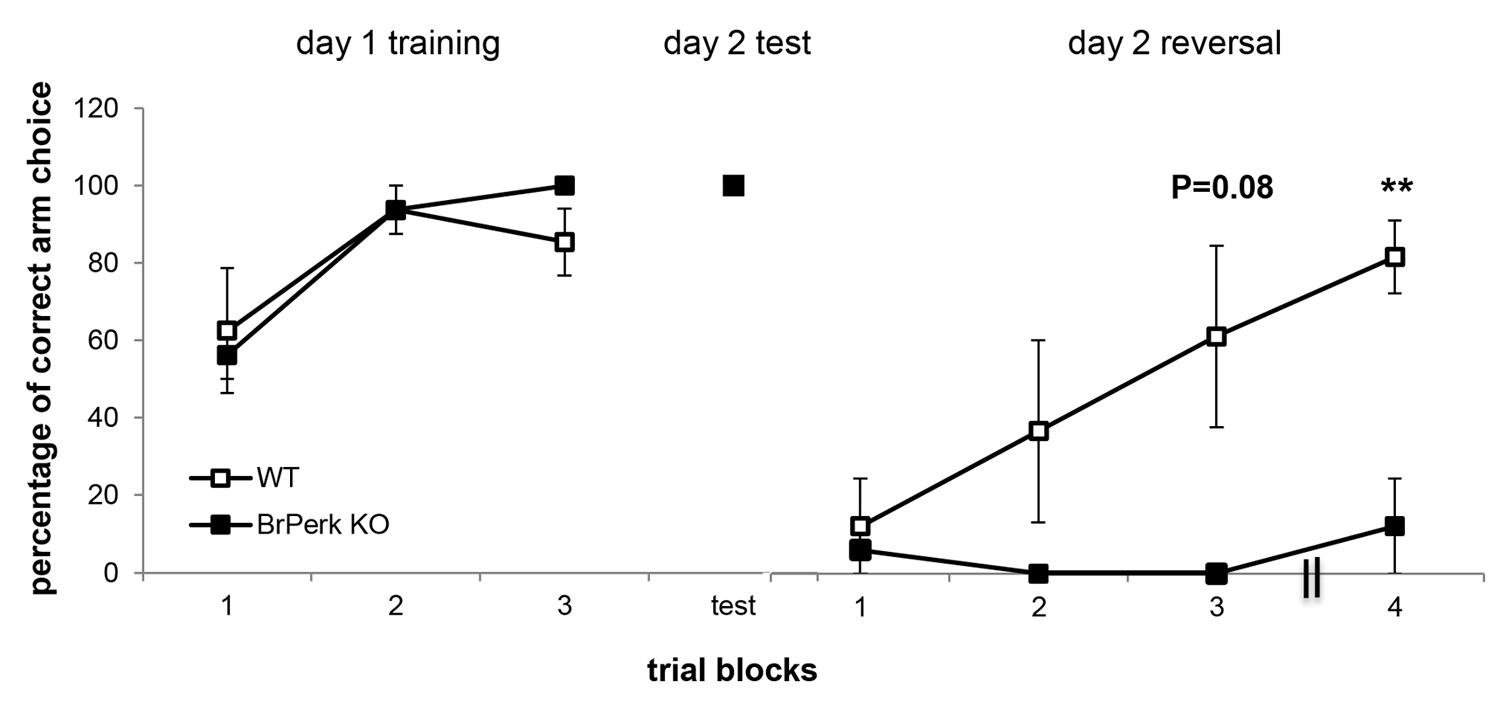

Supplement: S1 Fig — BrPerk KO mice exhibited normal spatial learning in the training session on day 1, and normal long-term memory in the test session on day 2 (WT: 4 out of 4 passed the test; BrPerk KO: 4 out of 5 passed the test). In the reversal session on day 2, when the platform was switched to the opposing arm, BrPerk KO mice exhibited impaired memory flexibility, as illustrated by the lower percentage of correct arm choice/block (WT n = 4; BrPerk KO n = 4; ** p<0.01. two-tailed student’s t-Test; 4 trials were included per block in the training and reversal session, 2 trials were performed in the test session). A retraining trial was performed between the third and fourth trial block in the reversal session, and the mice who never located the platform in its new location were guided onto it. (TIF) [file pone.0162766.s001.tif]

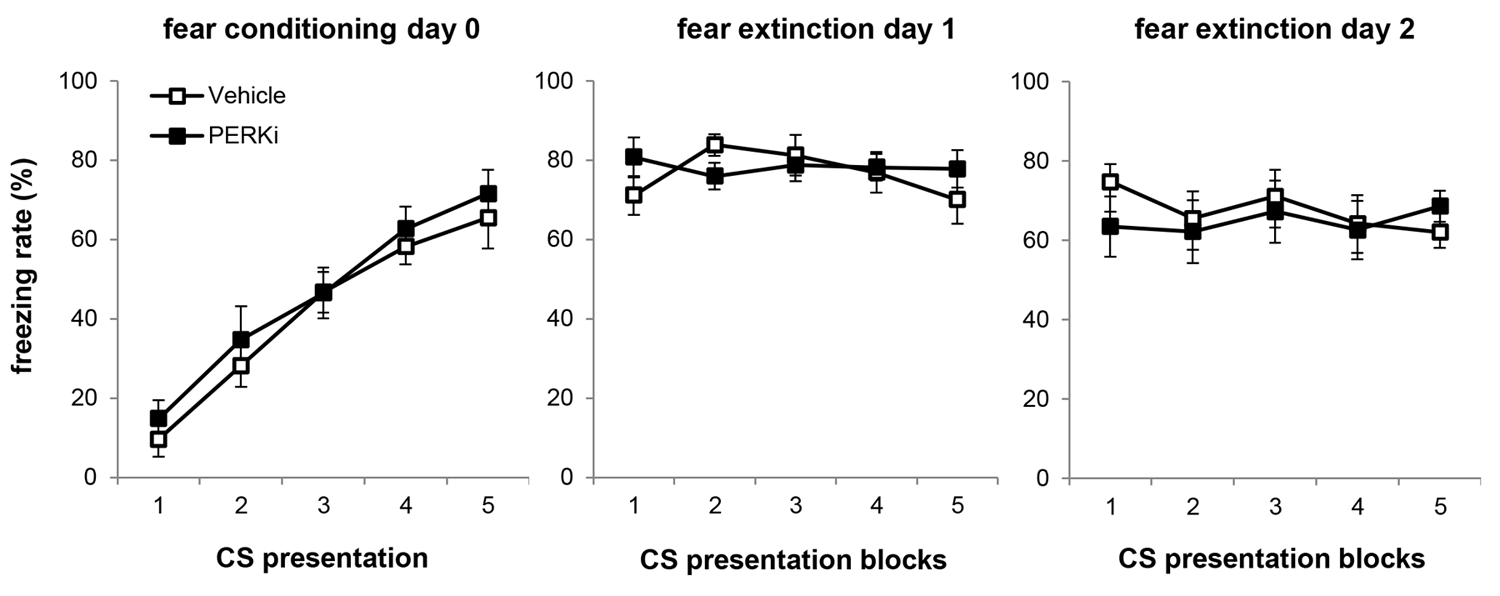

Supplement: S2 Fig — For mice received acute gavage administration without gavage acclimation, while both vehicle and PERKi group acquired conditioned fear on day 0, neither group exhibited fear extinction over the 2 day extinction training (Vehicle n = 10; PERKi n = 9). (TIF) [file pone.0162766.s002.tif]
