# Supplementary material for: PERK Regulates Working Memory and Protein Synthesis-Dependent Memory Flexibility
Source: PLoS One. 2016 Sep 14;11(9):e0162766. doi: 10.1371/journal.pone.0162766 (PMC5023101; doi:10.1371/journal.pone.0162766)
Supplement: S1 File — Material and method for Y-water maze reversal task. (DOCX) [file pone.0162766.s003.docx]

**Material and Method for S1 Fig.**

**Y-water maze reversal task**

Y-water maze reversal task was performed as previously described [19]. A symmetrical Y-maze with three identical arms (arms 35 cm long X 16cm high) was used and water was made opaque with non-toxic white paint to make the escape platform invisible. The experiment was consisted of 2 days, with training session on day 1 and test and reversal session on day 2. During the training session, each mouse was trained in 10-20 trials to locate a hidden escape platform placed at the end of one arm, with 60 s allowed in each trial. The starting arm remained the same during the experiment. Mice were considered passing the training once they made 5 successful trials in a row (a success is defined as the animal being able to get onto the platform at its first choice within 1 min). 24 hours later, the mice were returned to Y-water maze and tested for their long-term memory in 2-3 trials, with the platform staying in the same arm. Those who made all correct arm choices in the test trials were selected for the reversal session. Reversal session was carried out right after the test, with the escape platform switched to the opposing arm, and the mice’s ability to locate the new position of the platform was measured. The results for all sessions were manually recorded.
